# Tumor-Suppressive and Immunomodulating Activity of miR-30a-3p and miR-30e-3p in HNSCC Cells and Tumoroids

**DOI:** 10.3390/ijms241311178

**Published:** 2023-07-06

**Authors:** Ombline Conrad, Mickaël Burgy, Sophie Foppolo, Aude Jehl, Alicia Thiéry, Sébastien Guihard, Romain Vauchelles, Alain C. Jung, Jana Mourtada, Christine Macabre, Sonia Ledrappier, Marie-Pierre Chenard, Mihaela-Alina Onea, Aurélien Danic, Thomas Dourlhes, Claire Thibault, Philippe Schultz, Monique Dontenwill, Sophie Martin

**Affiliations:** 1 Laboratory of Bioimaging and Pathology, University of Strasbourg, UMR7021 CNRS, 67401 Illkirch, France; ombline.conrad@etu.unistra.fr (O.C.); m.burgy@icans.eu (M.B.); sophie.foppolo@unistra.fr (S.F.); monique.dontenwill@unistra.fr (M.D.); 2 Department of Medical Oncology, Institut de Cancérologie Strasbourg Europe, 67200 Strasbourg, France; 3 Department of Public Health, Institut de Cancérologie Strasbourg Europe, 67200 Strasbourg, France; 4 Department of Radiotherapy, Institut de Cancérologie Strasbourg Europe, 67200 Strasbourg, France; 5 Laboratory STREINTH, Inserm IRFAC U1113, Université de Strasbourg, 67200 Strasbourg, France; a.jung@icans.eu (A.C.J.);; 6 Laboratory of Tumor Biology, Institut de Cancérologie Strasbourg Europe, 67200 Strasbourg, France; 7 Department of Pathology, Strasbourg University Hospital, 67200 Strasbourg, France; marie-pierrette.chenard@chru-strasbourg.fr (M.-P.C.);; 8 Department of Otolaryngology and Cervico-Facial Surgery, Strasbourg University Hospital, 67200 Strasbourg, Francephilippe.schultz@chru-strasbourg.fr (P.S.)

**Keywords:** head and neck cancers, microRNA, tumoroid, biomarkers, phagocytosis

## Abstract

Head and neck squamous cell carcinomas (HNSCCs) are heterogeneous tumors, well known for their frequent relapsing nature. To counter recurrence, biomarkers for early diagnosis, prognosis, or treatment response prediction are urgently needed. miRNAs can profoundly impact normal physiology and enhance oncogenesis. Among all of the miRNAs, the miR-30 family is frequently downregulated in HNSCC. Here, we determined how levels of the 3p passenger strands of miR-30a and miR-30e affect tumor behavior and clarified their functional role in LA-HNSCC. In a retrospective study, levels of miR-30a-3p and miR-30e-3p were determined in 110 patients and correlated to overall survival, locoregional relapse, and distant metastasis. miR-30a/e-3p were expressed in HNSCC cell lines and HNSCC patient-derived tumoroids (PDTs) to investigate their effect on tumor cells and their microenvironment. Both miRNAs were found to have a prognosis value since low miR-30a/e-3p expression correlates to adverse prognosis and reduces overall survival. Low expression of miR-30a/e-3p is associated with a shorter time until locoregional relapse and a shorter time until metastasis, respectively. miR-30a/e-3p expression downregulates both TGF-βR1 and BMPR2 and attenuates the survival and motility of HNSCC. Results were confirmed in PDTs. Finally, secretomes of miR-30a/e-3p-transfected HNSCC activate M1-type macrophages, which exert stronger phagocytic activities toward tumor cells. miR-30a/e-3p expression can discriminate subgroups of LA-HNSCC patients with different prognosis, making them good candidates as prognostic biomarkers. Furthermore, by targeting members of the TGF-β family and generating an immune-permissive microenvironment, they may emerge as an alternative to anti-TGF-β drugs to use in combination with immune checkpoint inhibitors.

## 1. Introduction

Head and neck squamous cell carcinoma (HNSCC) develops from epithelial cells of the upper aerodigestive tract, including the oral cavity, pharynx, and larynx. HNSCC is the seventh most common cancer worldwide and is the leading cause of cancer death with approximately 900,000 new cases per year and 450,000 deaths estimated [1]. Despite its severity and increasing prevalence within society (+25% in the last decade), there is little awareness of this cancer from the public. The main risk factors are tobacco and alcohol consumption. HNSCC from the oropharynx is mainly due to human papillomavirus (HPV) infection [2]. Patients diagnosed in the early stages of the disease have an 80–90% survival rate with good quality of life after single-modality treatment, mainly surgery or radiotherapy. Unfortunately, most tumors (60% of newly diagnosed patients) are not diagnosed until they are locally advanced (LA-HNSCC: stage III or IV of UICC 8th edition) or metastasized. Late detection limits the effectiveness of combined modality therapy involving a primary surgery followed by radiotherapy and/or chemotherapy [3,4,5]. High recurrence rates (secondary primary tumors, locoregional recurrence, and distant metastasis occurring in 20–30% of patients) hamper the success of multimodal therapeutic procedures leading to poor prognosis with an overall 5-year survival rate of <50% [6]. In clinicopathological practice, it is difficult to assign patients into classes of risk since no reliable biomarkers are available to predict the outcome of HNSCC. In contrast to other cancers, multidisciplinary treatment decisions for patients suffering from HNSCC are still based on TNM staging and anatomical localization.

HNSCC is well known for its heterogeneity, anatomical diversity, and relapsing nature. To counter recurrence and resistance, but also to identify biomarkers for early diagnosis, prognosis, or treatment response prediction, an improved understanding and characterization of these tumors are needed. The human genome project showed that non-coding RNAs, such as miRNAs, are transcribed and function in normal and pathological cells [7,8]. miRNAs are endogenous, small non-coding RNAs of 19–25 nucleotides. They fine-tune gene expression by binding to the 3′ untranslated region of the target mRNA, leading to mRNA degradation or mRNA translational repression [9]. Over the past decade, several studies have shown that dysregulation of miRNAs can profoundly impact normal physiology and enhance oncogenesis [10,11]. Analysis of miRNA signature and TCGA has revealed that the miR-30 family is frequently downregulated in HNSCC [12,13]. The miR-30 family contains six miRNAs (miR-30a/b/c1/c2/d/e) encoded by six genes located on three distinct loci of the human genome (chromosome 1 for miR-30e/c1, chromosome 6 for miR-30a/c2, and chromosome 8 for miR-30d/b) [14]. Each miRNA duplex consists of two mature complementary miRNA strands: the guide strand 5p and the passenger strand 3p. If guide strands of the miR-30 family share the same SEED (mRNA-targeting) sequence (5′-GUAAACA-3′), passenger strands have two different SEED sequences: 5′-UUUCAGU-3′ for miR-30a/d/e and 5′-UGGGAG-3′ for miR-30b/c. Within this family, miR-30a and miR-30e have been described as important anti-oncomiR (or tumor suppressor miRNAs) [12,13,15,16]. Although both miRNAs have been reported to be consistently downregulated in HNSCC compared to adjacent normal tissue [12,13,17,18,19,20], no data are available on the impact and consequences of variability in their expression on LA-HNSCC tumor behavior and progression. Moreover, functional studies have focused mainly on the 5p guide strand and less on the 3p strand, which was thought to be only passenger and always eliminated. We know now that both strands are functional and may or may not have similar functions. Because miR-30-5p is downregulated in HNSCC compared to adjacent normal tissue and act as a tumor suppressor [13], the role of its passenger strand miR-30-3p was explored. How levels of the 3p strands of miR-30a and miR-30e affect tumor behavior and their functional role in LA-HNSCC using both HNSCC cell lines and patient-derived tumoroids (PDT) were determined. The use of PDTs known to recapitulate tumor microenvironment signaling by permitting cell–cell contacts, extracellular matrix interactions, and cell signaling further strengthens our data, showing the ability of miR-30a/e-3p to stimulate PDT immune infiltration. We determine here that reduced miR-30a/e-3p levels in primary tumors are responsible for poor prognoses. We show for the first time that while the disappearance of miR-30a-3p is associated with locoregional relapse, the disappearance of miR-30e-3p is associated with metastatic relapse. Tumor-suppressive activity is characterized by a reduction in cell proliferation and motility and an induction of apoptosis in cell lines, as well as in patient-derived tumoroids. This is linked to the ability of miR-30a/e-p to inhibit two never-before-described targets, TGFβR1 and BMPR2, in tumor cells. We also describe here for the first time a modification of the peritumoral secretome linked to miR-30a/e-3p expression that promotes macrophage phagocytic activity. Overall, these new data provide a better understanding of how miR-30a/e-3p exert their antitumor activity in HNSCC.

## 2. Results

### 2.1. Expression of miR-30-3p in HNSCC Tumors Is Correlated with Good Prognosis and Is Associated with Increased Overall Survival

**Figure 1:**
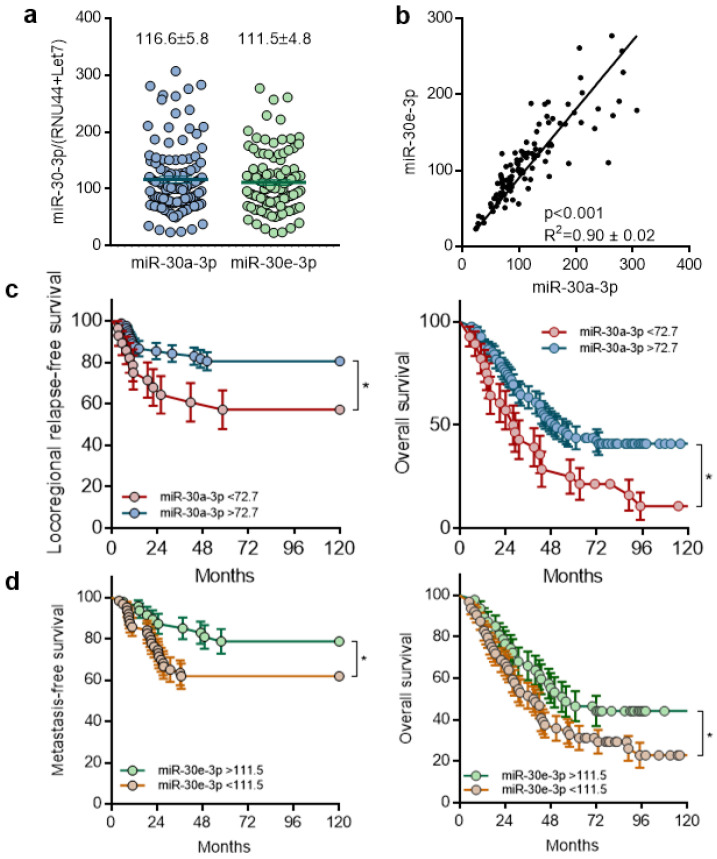
Expression of miR-30-3p in HNSCC tumors is correlated with good prognosis and is associated with increased overall survival. (**a**) miR-30a-3p and miR-30e-3p expression was determined by RT-qPCR in HNSCC tumor samples (*n* = 110). The line within the bar represents the mean value and “●” represents individual data point. (**b**) Correlation of miR-30a-3p and miR-30e-3p expression in HNSCC tumor samples (*n* = 110). (**c**) Kaplan–Meier analysis of miR-30a-3p expression in HNSCC tumor samples correlated to locoregional relapse-free survival and overall survival (*n* = 110; * *p* < 0.05). (**d**) Kaplan–Meier analysis of miR-30e-3p expression in HNSCC tumor samples correlated to metastasis-free survival and overall survival (*n* = 110; * *p* < 0.05).

To explore the role of miR-30a-3p and miR-30e-3p in HNSCC, a cohort of 110 patients with stage III-IV HPV-negative primary HNSCC (mean age 60 ± 10, range 36–84 year) was enrolled in this study. miR-30a-3p and miR-30e-3p are expressed at similar levels in LA-HNSCC (116.6 ± 5.8 and 111.5 ± 4.8, respectively, Figure 1a) and are significantly positively correlated with each other (R2 = 0.90, *p* < 0.001, Figure 1b). Patients were stratified according to miR-30a-3p and miR-30e-3p expression. The threshold between high (*n* = 82) and low (*n* = 28) expression was set at the level of the first quartile for miR-30a-3p. The threshold between high (*n* = 46) and low (*n* = 64) expression was set at the average (111.5) of miR-30e-3p. Kaplan–Meier analysis of locoregional relapse-free survival (LRFS), metastasis-free survival (MFS), and overall survival (OS) was performed. Primary endpoints were metastatic disease and locoregional recurrence free survival at 3 years after surgery. miR-30a-3p and miR-30e-3p expression could statistically discriminate two subgroups of patients. Both miRNAs were found to have a prognosis value since low miR-30a-3p and low miR-30e-3p expressions correlated with adverse prognosis (Figure 1c,d left) and poorer overall survival (Figure 1c,d right). In addition, low expression of miR-30a-3p was associated with a shorter time until locoregional relapse (Figure 1c left), and low expression of miR-30e-3p was associated with a shorter time until metastasis (Figure 1d left). Altogether, these data suggest that loss of miR-30a-3p and miR-30e-3p expression is correlated with tumor recurrence and poor prognosis.

### 2.2. Expression of miR-30-3p in HNSCC Cells Reduces Survival and Slows down Evasion

**Figure 2:**
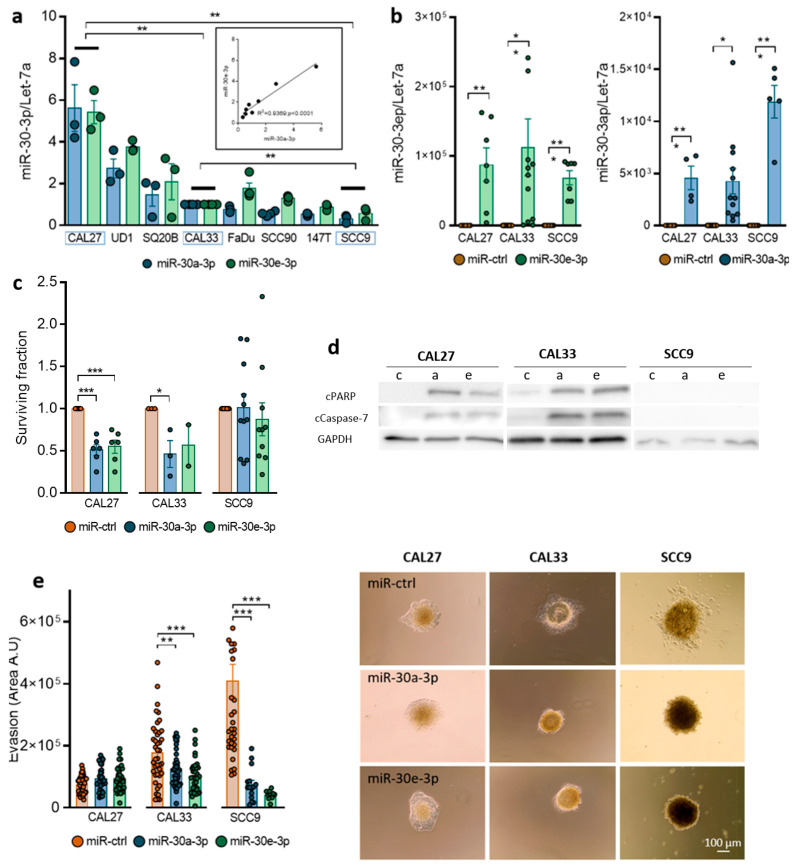
Expression of miR-30-3p in HNSCC cells reduces survival and slows down evasion. (**a**) miR-30a-3p and miR-30e-3p expression was determined by RT-qPCR in 8 HNSCC cell lines (*n* = 3, ** *p* < 0.01). (**b**) miR-30a-3p and miR-30e-3p expression was determined by RT-qPCR in transfected CAL27, CAL33, and SCC9 cell lines (*n* = 5–12, * *p* < 0.05 and ** *p* < 0.01). (**c**) Survival fraction of CAL27, CAL33, and SCC9 cell lines overexpressing miR-30a-3p or miR-30e-3p (*n* = 3–11, * *p* < 0.05 and *** *p* < 0.001). (**d**) Western blot analysis of CAL27, CAL33 and SCC9 cell lines overexpressing miR-30a-3p or miR-30e-3p (c for miR-ctrl, a for miR-30a-3p and e for miR-30e-3p, *n* = 3) (**e**) Evasion of CAL27, CAL33, and SCC9 cell lines overexpressing miR-30a-3p or miR-30e-3p (*n* = 15–49 spheroids, ** *p* < 0.01 and *** *p* < 0.001).

miR-30a-3p and miR-30e-3p were heterogeneously expressed among eight HNSCC cell lines studied (Figure 2a). As reported for LA-HNSCC, the expressions of both miRNAs are positively correlated (R2 = 0.9369, *p* < 0.001, Figure 2a). To determine underlying mechanisms that could explain relapses and decreased overall survival, miR-30a-3p and miR-30e-3p were overexpressed (Figure 2b) in three cell lines, showing high (CAL27), intermediate (CAL33), and low (SCC9) levels of each miRNA. Overexpression of both miRNAs only mildly affects the growth of cells (see Supplementary Data S1) but significantly reduces clonogenic survival of CAL27 and CAL33 without affecting SCC9 (Figure 2c). Cleavage of PARP and caspase-7 was detected in CAL27 and CAL33 cells, suggesting that reduced survival observed in both cell lines but not in SCC9 cells was most likely due to an induction of apoptosis (Figure 2d). The overexpression of both miRNAs reduces tumor cell evasion of CAL33 and SCC9 without affecting CAL27 (Figure 2c). Altogether, the data show that miR-30a-3p and miR-3e-3p alter survival and/or evasion of tumor cells without altering their growth capacity.

### 2.3. TGFBR1 and BMPR2 Are the Main Effector Targets of miR-30-3p in HNSCC

**Figure 3:**
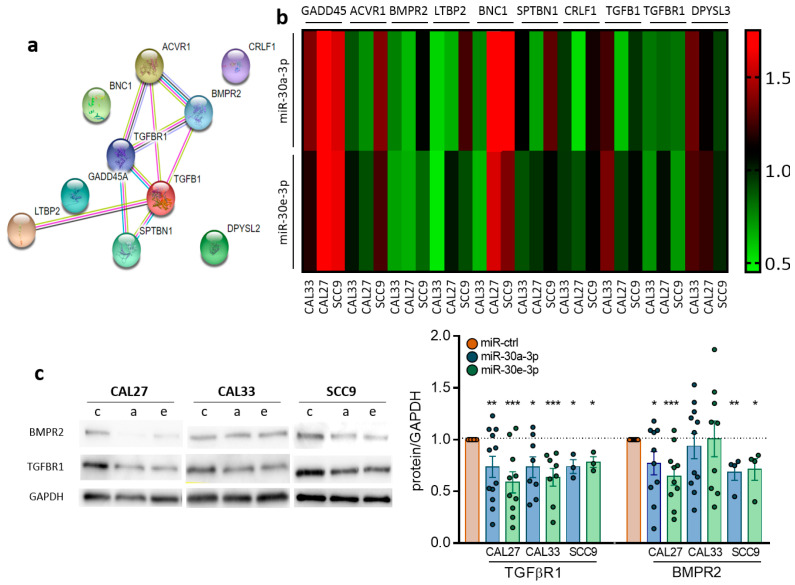

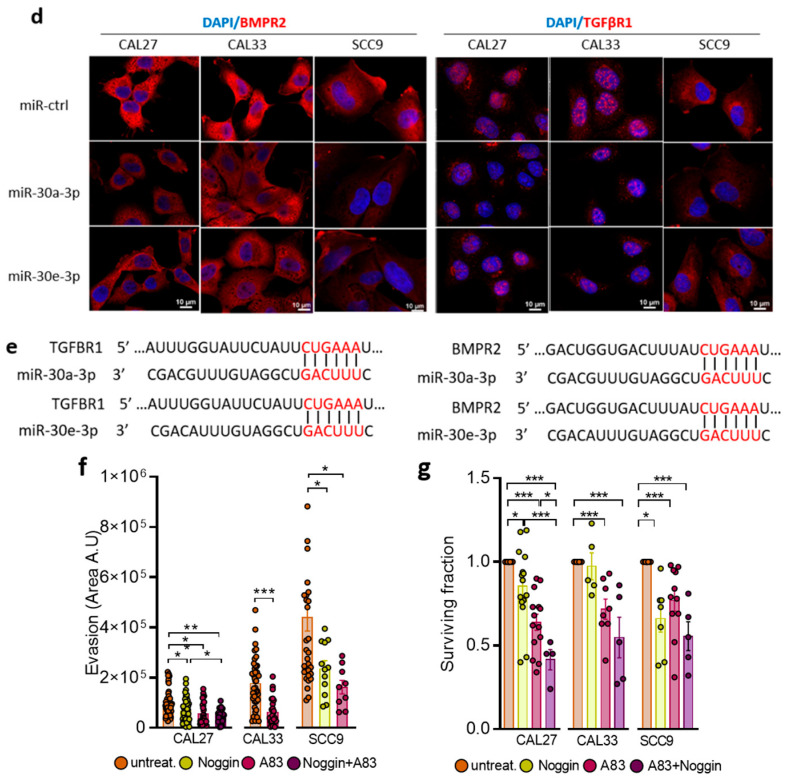
TGFBR1 and BMPR2 are the main effector targets of miR-30-3p in HNSCC. (**a**) Predicted interaction between target proteins was analyzed using STRING database. (**b**) Heatmap representing predicted target gene expression was determined by RT-qPCR in HNSCC cell lines (*n* = 4–7). (**c**) Western blot analysis of CAL27, CAL33, and SCC9 cell lines overexpressing miR-30a-3p or miR-30e-3p (c for miR-ctrl, a for miR-30a-3p and e for miR-30e-3p, *n* = 3–12, * *p* < 0.05, ** *p* < 0.01 and *** *p* < 0.001). (**d**) Immunofluorescence analysis by confocal microscopy of HNSCC cell lines overexpressing miR-30a-3p or miR-30e-3p (*n* = 3–4). (**e**) Predicted pairing region between miR-30a-3p or miR-30e-3p and TGFBR1 (position 812–818) and BMPR2 (position 1411–1417). (**f**) Evasion of CAL27, CAL33, and SCC9 cell lines with TGF-β or BMP inhibitors (*n* = 16–49 spheroids, * *p* < 0.05, ** *p* < 0.01 and *** *p* < 0.001). (**g**) Survival fraction of CAL27, CAL33, and SCC9 cell lines with TGF-β or BMP inhibitors (*n* = 9–12, * *p* < 0.05 and *** *p* < 0.001).

To identify the network of miR-30a-3p and miR-30e-3p mRNA targets, we explored whether reduced expression of both miRNAs was anticorrelated with mRNAs of potential biological importance in cancer. Linear regression analysis was performed between both miRNAs and genome-wide mRNA expression levels obtained from RNA-seq tumor specimens in the TCGA dataset (TCGA-HNSC project of the databank cBio Cancer Genomic Portal). Ten mRNAs were negatively correlated with both miRNAs: DPYSL3, TGFBR1, TGFB1, CRLF1, SPTBN1, BNC1, LTBP2, BMPR2, ACVR1, and GADD45A. Six of them interact with each other and belong to or are closely related to the TGFβ signaling pathway as shown in STRING analysis: ACVR1, TGFBR1, TGFB1, SPTBN1, LTBP2, and BMPR2 (Figure 3a). Although most of the genes are indeed downregulated in HNSCC cells expressing miR-30a-3p or miR-30e-3p, as confirmed by RT-qPCR, some of them are overexpressed in either cell lines or in response to either miRNA (such as GADD45, ACVR1, LTBP2, BNC1, SPTBN1, and DPYSL3 in SCC9, Figure 3b). As TGFBR1 and BMPR2 are both downregulated at the RNA level in all cell lines by both miRNAs (Figure 3b), we focused the rest of the study on the common targets of miR-30a-3p and miR-30e-3p. Downregulation was confirmed at the protein level by Western blot and immunofluorescence analysis for TGFBR1 in CAL27, CAL33, and SCC9 and for BMPR2 in CAL27 and SCC9 but not in CAL33 (Figure 3c,d). Using TargetScan, we predicted SEED sequences pairing of miRNAs with TGFBR1 and BMPR2 (Figure 3e). TGFBR1 and BMPR2 signaling pathways were inhibited using A8301 and Noggin, respectively, to confirm their involvement in the in vitro relapse features of miR-30a-3p and miR-30e-3p. A8301 is a kinase inhibitor of TGFBR1 that blocks Smad2 phosphorylation and inhibits TGFb signaling. Noggin is an antagonist of BMPs that prevents them from activating the BMP pathway. A8301 significantly reduces both evasive capacity and clonogenic survival in all cell lines investigated (Figure 3f,g). Similar results were obtained for Noggin in CAL27 and SCC9 but not CAL33, which is consistent with the fact that BMPR2 was not affected by miR-30-3p in those cells (Figure 3c,d). Combining both drugs in CAL27, but not in SCC9, resulted in a significantly greater reduction in evasion and survival than each drug alone (Figure 3f,g). Growth was only mildly affected by both drugs. Altogether, the data show that pharmacological inhibition of the TGFBR1 and BMPR2 signaling pathways phenocopies the overexpression of miR-30a-3p and miR-30e-3p.

### 2.4. Overexpression of miR-30-3p Slows down HNSCC Patient-Derived Tumoroid Growth and Development

**Figure 4:**
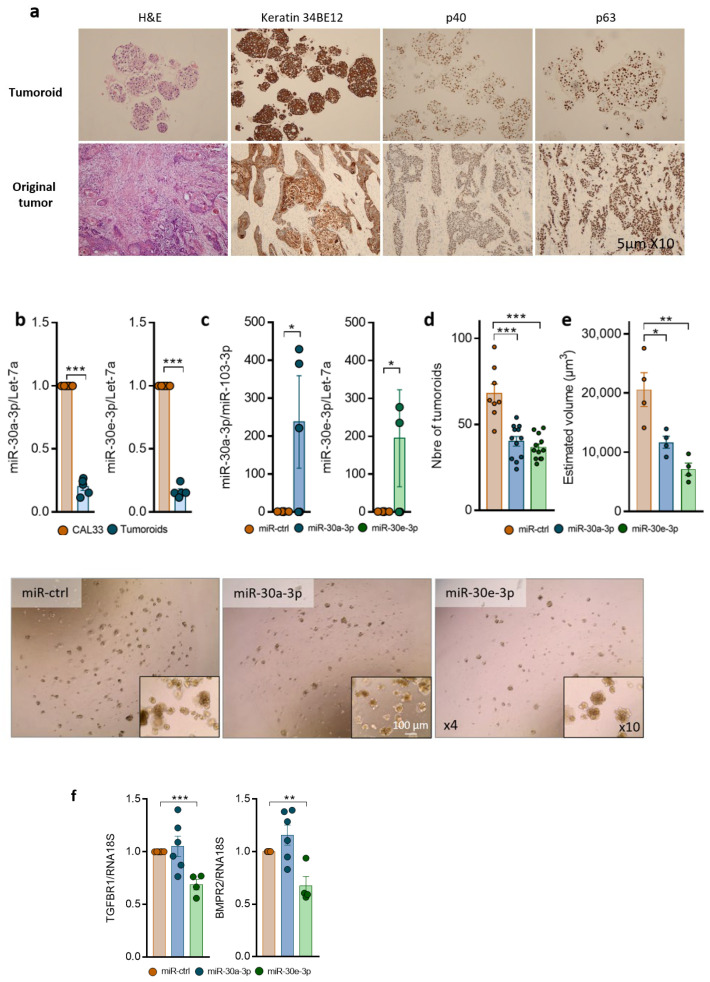

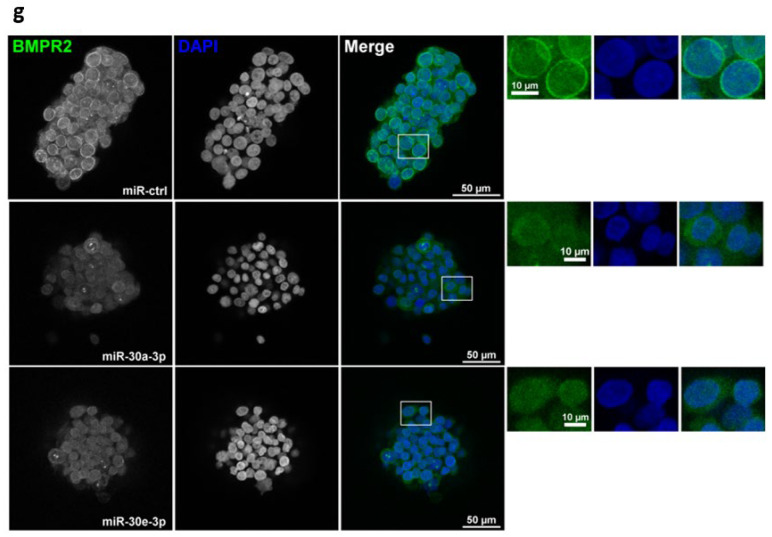
Overexpression of miR-30-3p slows down HNSCC patient-derived tumoroid growth and development. (**a**) Immunohistochemical staining of Keratin 34BE12, p40, p63, and hematoxylin and eosin staining in tumoroid versus the original tumor. (**b**) miR-30a-3p and miR-30e-3p expression was determined by RT-qPCR in tumoroids (*n* = 5, *** *p* < 0.001). (**c**) miR-30a-3p and miR-30e-3p expression was determined by RT-qPCR in tumoroids post-transfection (*n* = 4–5, * *p* < 0.05) (**d**) Number of tumoroids remaining at day 6 post-transfection with mir-30a-3p or miR-30e-3p (*n* = 10–20 tumoroids, *** *p* < 0.001). (**e**) Volume of tumoroids at day 6 transfected with miR-30a-3p or miR-30e-3p. Estimated volume = (4/3) × π (d1/2 × d2/2 × d3/3) (*n* = 10–20 tumoroids, * *p* < 0.05 and ** *p* < 0.01). (**f**) TGFBR1 and BMPR2 expressions were determined by RT-qPCR in tumoroids (*n* = 4–6, ** *p* < 0.01 and *** *p* < 0.001). (**g**) BMPR2 expressions were determined by immunostaining in tumoroids (*n* = 3).

Patient-derived tumoroids are a novel relevant model to study the mechanisms of cancer progression and resistance as they maintain key features of the tumors from which they originate. Tumoroids were derived from a 68-year-old man with HPV-negative squamous cell carcinoma of the anterior oral floor, histologically described as T3N0M0. Histological examination of the primary tumor and corresponding tumoroids showed that tumoroids recapitulate cell architecture, differentiation, and heterogeneity of the primary tumors (Figure 4a). p40 (Delta Np63), p63, Keratin34BE12 (Figure 4a), and Ker 5/6 expressions were similar in the primary tumor and the tumoroid, confirming the epithelial and squamous cell carcinoma origin. Analysis of miR-30a-3p and miR-30e-3p showed that they are expressed at low levels when compared to the CAL33 cell line (Figure 4b) and maintained low expression throughout the culture. Overexpression of miR-30a-3p and miR-30e-3p (Figure 4c) decreased the number of tumoroids (Figure 4d), as well as their volume (Figure 4e), when compared to the control, confirming the tumor-suppressive role of miR-30-3p. As observed in vitro, both BMPR2 and TGFBR1 are inhibited at the mRNA level in tumoroids when miR-30e-3p is overexpressed (Figure 4f). Immunofluorescence staining followed by confocal analysis showed that BMPR2 was localized to the plasma membrane of cells forming tumoroids under miR-ctrl conditions. Expression of miR-30a/e-3p was associated with the loss of the membrane distribution of BMPR2, as well as the decrease in the intensity of BMPR2 staining (Figure 4g). Similar data could be obtained with TGFBR1 and in tumoroids from other patients. All these data suggest that miR-30a-3p and miR-30e-3p expression by tumor cells prevent prevents tumoroid growth and development.

### 2.5. miR-30a-3p and miR-30e-3p Influence Macrophages’ Immune Response to HNSCC Cells

**Figure 5:**
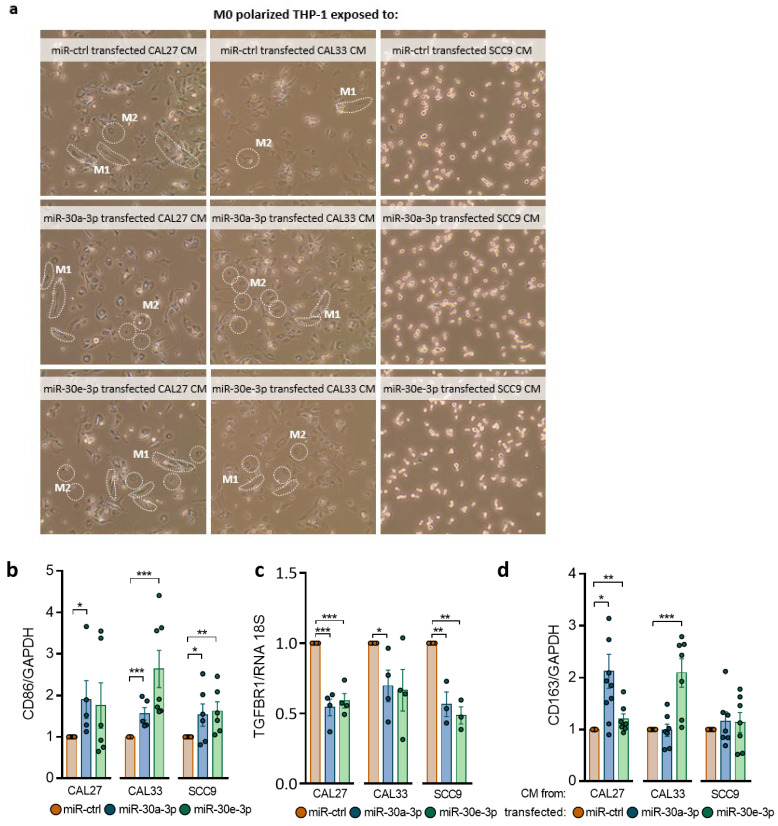

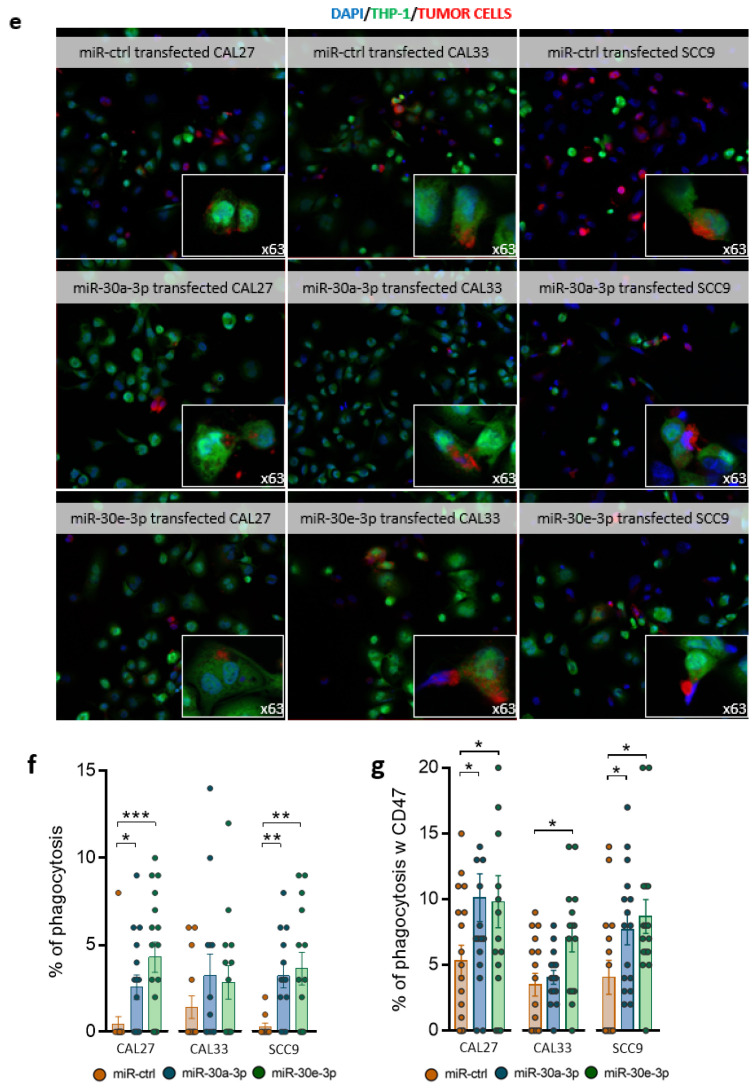
miR-30a-3p and miR-30e-3p influence macrophages’ immune response to HNSCC cells. (**a**) Images of M0-polarized THP-1 exposed to CAL27, CAL33, SCC9 overexpressing miR-30a-3p and miR-30e-3p conditioned media in phase-contrast microscopy (×10). (**b**) Western blot analysis of CD86 in M0-polarized THP-1 exposed to CAL27, CAL33, and SCC9 overexpressing miR-30a-3p and miR-30e-3p conditioned media (*n* = 7–10, * *p* < 0.05, ** *p* < 0.01, *** *p* < 0.001). (**c**) TGFBR1 expression was determined by RT-qPCR in M0-polarized THP-1 exposed to CAL27, CAL33, SCC9 overexpressing miR-30a-3p and miR-30e-3p conditioned media (*n* = 3–4, * *p* < 0.05, ** *p* < 0.01, *** *p* < 0.001). (**d**) Western blot analysis of CD163 in M0-polarized THP-1 exposed to CAL27, CAL33, and SCC9 overexpressing miR-30a-3p and miR-30e-3p conditioned media (*n* = 8–10, * *p* < 0.05, ** *p* < 0.01, *** *p* < 0.001). (**e**) Images of MO-polarized THP-1 cells (green) phagocytosing miR-30a-3p or miR-30e-3p-transfected HNSCC cells (red) in confocal microscopy 20× and 63×. (**f**) Percentage of phagocytosis, calculated as the number of M0-polarized THP-1 cells phagocytosing transfected HNSCC cells/total number of M0-polarized THP-1 × 100 (*n* = 3–4, * *p* < 0.05, ** *p* < 0.01, *** *p* < 0.001). (**g**) Percentage of phagocytosis with antibody antiCD47, calculated as the number of M0-polarized THP-1 cells phagocytosing transfected HNSCC cells/total number of M0-polarized THP-1 × 100 (*n* = 10–18, * *p* < 0.05).

TGFβ signaling has key roles in cancer progression. Studies including the TCGA database show a positive correlation between high TGFβ1 expression and/or TGFβR1 signaling and macrophages (mainly M2-like) infiltrate in HNSCC [21]. M2-like macrophages are endowed with a repertoire of tumor-promoting capabilities, among which immuno-suppression is known to support tumor progression. Since macrophages were reported to be the most abundant immune cell type in the tumor microenvironment of OSCC, we investigated how they might be affected by secretomes of miR-30a-3p or miR-30e-3p-transfected tumor cells. M0 macrophages polarize into CD86+ M1-like macrophages (Figure 5a,b) when exposed to a conditioned medium (CM). Incubation with the CM inhibited TGFBR1 expression in macrophages, which likely skewed macrophages to the M1 end (Figure 5c). Expression of CD163+, a marker of M2-like macrophages, was detected in miR-30a-3p and miR-30e-3p-transfected CAL27 and the miR-30e-3p-transfected CAL33 CM. BMPR2 expression remained unaffected in macrophages incubated in the CM from miR-30-3p-transfected-CAL27 and -SCC9 and increased in the CM from miR-30-3p-transfected CAL33 (Supplementary Data S2a). It is worth noting that miR-30a-3p and miR-30e-3p levels were upregulated in M0 macrophages exposed to miR-30a/e-3p-transfected CAL27, CAL33, and SCC9 CM (Supplementary Data S2b,c). However, the levels achieved are far lower than those obtained by direct transfection (see Figure 2b). We then co-cultured macrophages (green staining) with tumor cells (red staining). We found increased phagocytic activities of macrophages towards tumor cells expressing high levels of miR-30a-3p or miR-30e-3p compared to control conditions (Figure 5e,f). HNSCC and OSCC often overexpress the “don’t eat me” signal CD47/SIRPα [22]. In vitro phagocytosis of tumor cells expressing miR-30a-3p or miR-30e-3p by macrophages was significantly enhanced after CD47/SIRPα blockade (Figure 5g). Altogether, the data suggest that miR-30a-3p and miR-30e-3p expression by tumor cells generate an immune-permissive microenvironment favoring M1-like macrophage polarization and tumor cell phagocytosis.

## 3. Discussion

Until very recently, the miR-30 family was not extensively studied in HNSCC in contrast to other cancers. This changed with the comprehensive meta-analysis conducted by Zeljic and colleagues in 2018 [23]. By comparing seven published miRNA expression profiling [17,20,24,25], the authors showed that both 5p and 3p strands of miR-30a were consistently downregulated in oral cancers when compared to matched noncancerous tissue [23]. In the search for miRNA of potential importance in HNSCC, Saleh and colleagues identified miR-30(a,b,d,e)-5p as tumor suppressors and potential therapeutic targets [13]. However, the passenger strands of miR-30a and miR-30e still remain poorly studied in HNSCC. Using TCGA-HNSC data, Minemura and colleagues confirmed that miR-30a-3p and miR-30e-3p were downregulated in HNSCC [12]. Patients with low expression of miR-30e-3p had significantly poorer prognosis compared to those with a high expression [26]. In accordance, we show here that low expression of both miR-30a-3p and miR-30e-3p was associated with reduced overall survival. However, we went further by showing that miR-30a-3p and miR-30e-3p could discriminate between patients progressing to locoregional relapse or metastatic recurrence. Whereas loss of miR-30a-3p seems to be associated with locoregional recurrence, metastatic prone tumors show rather low miR-30e-3p levels. Our results reinforce data showing the potential of miR-30a/e-3p to become diagnostic and prognostic biomarkers and highlight for the first time their ability to predict local or systemic recurrence of HNSCC.

Less studied than their 5p homologues, the functional characteristics of these two miRNAs are poorly characterized and remain to be discovered. Expression of miR-30a/e-3p significantly attenuates the survival and motility of HNSCC cell lines without impacting their ability to proliferate, confirming their tumor-suppressive capacity. The expression of miR-30e-3p attenuates migration and invasive abilities in SAS and Sa3 HNSCC cell lines but also significantly affects their proliferation [26]. Such differences could be related to the genes targeted by these miRNAs that might differ depending on the cell context or tumor type. One single miRNA can regulate hundreds of mRNAs and thus govern an entire expression network. Without being exhaustive, targets of the 5p guide strands of miR-30a/e identified so far are involved in the regulation of cell-cycle progression/proliferation/growth (p21, p27 [27], cyclin D [10], K-Ras [16], EGFR [28], MET [13], IGF1R [13], IRS1 [13,29], MYBL2 [30], MTDH/AEG1 [31]), DNA methylation (DNMT3 [15,19,32] and DNMT1 [33]), survival (BIRC6 [34], AVEN [34], Bcl-2 [35], BCL-xL, MDM2, p53 [28]), autophagy [36] and chemoresistance (HIF [37,38], MDR1, [39] ATG5 [40], Beclin-1 [40,41]), EMT (SNAIL [29,42], SLUG, ZEB2 [43,44]) migration/invasion (α2 [45], α5 [46], α6 [13] β3 [46,47], ADAM19, PAI-1 [13,48]), or radioresistance [49]. Because SEED sequences differ between the 5p guide strand and the 3p passenger strand, we searched for novel targets of miR-30a/e-3p. Performing linear regression analysis between both miRNAs and genome-wide mRNA from the TCGA datasets, we showed that most genes identified as potential targets belong to/or are closely related to the TGF-β signaling pathway. This pathway is found to be the most enriched signaling pathway among the 24 KEGG pathways identified in the oral cancer miRNA meta-signature conducted by Zeljic and colleagues [23].

TGFBR1 (TGF-βRI) and BMPR2 are two never-before-described targets of miR-30a/e-3p. Inhibition of both pathways significantly attenuates survival and evasive potential of cells and phenocopies miR-30a/e-3p. TGFBR1 and BMPR2 have been previously described as important players in the carcinogenesis and tumorigenesis of several cancers, including HNSCC [50,51,52,53]. TGF-βRI is mutated in 19% of HNSCC patients with metastasis [54]. TGF-β has also been reported to silence miR-30a-5p through the STAT3/MALAT1 pathway in HNSCC favoring tumor growth [55]. Although less studied in HNSCC, BMP signaling or high expressions of BMPR1A and BMPR2 have been observed in metastatic lower-lip SCC [52] and result in resistance to cetuximab [56] or confer proliferation and survival capabilities to OSCC [57]. Besides its effect on tumor cells, TGF-β signaling promotes inflammation and immune suppression in the tumor microenvironment (TME). Tumor-associated macrophages (TAMs) being the major component of the TME of HNSCC [58], we evaluated how the secretomes of miR-30a/e-3p-expressing tumor cells might affect TAM. Secretomes (containing the miR-30a/e-3p themselves) polarize macrophages mainly into M1-like TAMs most likely through the inhibition of TGF-β signaling. M1-like TAMs are potent effector cells in the killing of tumor cells, which is consistent with our observation of an increase in phagocytic activity of macrophages exposed to miR-30a/e-3p secretomes towards tumor cells. Thus, restoring M1-like TAM phagocytic activities towards tumor cells may contribute to improved disease-free survival and progression-free survival observed in tumors with high levels of miR-30a/e-3p. To date, only a low number of CD163+ M2-like macrophages was correlated to better DFS and PFS [59]. Our work shows that therapy that restores miR-30a/e-3p levels in tumor cells could attenuate tumor progression by acting on both the tumor and its immune microenvironment.

A wealth of preclinical findings has demonstrated that targeting TGF-β signaling is a promising means of exerting antitumor activity, and several classes of TGF-β inhibitors have been developed and tested in clinical trials [60,61]. None of them are currently approved for cancer therapy, mainly because inhibition of TGF-β signaling does not lead to clinical meaningful tumor regression, precluding their use in single therapy. The focus is now on anti-TGF-β combined with other chemotherapy or on bifunctional fusion proteins targeting TGF-β and PD-L1, for example, Bintrafusp alpha (M7824, recently discontinued prematurely by GSK-Merck) or SHR 1701 [62]. We propose here a new option in the targeting of TGF-β signaling based on the restoration of the expression of miR-30a/e-3p. The multi-targeting capacity of miR-30a/e-3p makes them particularly effective as antitumor agents as validated in our preclinical study using tumoroids. Tumoroids have emerged recently as robust preclinical models because they are derived from patients and recapitulate the original tumor heterogeneity, as well as its resistance to therapy [63,64]. miR-30a-3p and miR-30e-3p overexpression attenuates both TGBR1 and BMPR2 levels and decreases tumoroid number and volume. Together, data validate the tumoroid as a reliable preclinical model that is suitable for screening new therapies. Further studies using immunocompetent tumoroids are needed to validate the influence of the miR-30a/e-3p secretome on the tumor immune landscape and the relevance of targeting miR-30a/e-3p coupled with ICIs or anti-CD47s, such as magrolimab.

## 4. Materials and Methods

### 4.1. Human Tissue Samples

All tumor specimens (N = 110) were collected during the initial surgery, stored, and used with the patients’ informed consent. Patients from the north-east region of France underwent initial surgical resection of their localized head and neck squamous cell carcinoma (HNSCC) between 2003 and 2013 at the St Barbe Clinic (Strasbourg, France), followed by post-operative radiotherapy or chemoradiotherapy at the Paul Strauss Cancer Center (Strasbourg, France) or the Civil Hospitals of Colmar or Mulhouse. All of the tumors were squamous cell carcinomas (SCCs). The inclusion criteria were tumor localization (hypopharynx, oropharynx or oral cavity, HPV-negative) ≥T3 and/or ≥N2a with no clinical or radiographic evidence of distant metastases. Primary endpoints were metastatic disease and loco-regional recurrence free survival at 3 years after surgery. Secondary endpoints included overall survival (OS), defined as the time from surgery to the date of death or last follow-up. Recorded variables included age, Eastern Cooperative Oncology Group (ECOG) and Karnofsky Performance Score (KPS), comorbidities (Charlson Comorbidity Index), tumor stage, chemotherapy regimen, smoking and alcohol consumption, and follow-up data (survival data, biological parameters, and nutritional characteristics). For detailed patient demographics, see Supplementary Table S1 [65].

### 4.2. Cell Culture, Transfection, and Drugs

CAL27, CAL33, SCC9, and THP-1 cell lines were purchased from ATCC^®^ and DSMZ (authenticated by STR profiling). All cell lines tested negative for mycoplasma contamination. CAL27 and CAL33 were grown in DMEM (PAN Biotech, Dutscher SAS, Brumath, France), supplemented with 0.5 mM sodium pyruvate and 10% heat-inactivated FBS (Gibco, Dutscher SAS, Brumath, France). SCC9 was grown in DMEM-F12 (PAN Biotech), supplemented with 2.5 mM ultra-glutamine, 15 mM HEPES, 400 ng/mL hydrocortisone (Sigma-Aldrich, St Quentin Fallavier, France), and 10% FBS (Gibco). THP-1 cells were grown in RPMI-1640 (Sigma-Aldrich) supplemented with 10% heat-inactivated FBS (Gibco). To overexpress miR-30a-3p and miR-30e-3p, cells were transfected for 72 h either by 10 nM miR-30a-3p (Qiagen, Hilden, Germany) or miR-30e-3p (Qiagen) using HiPerFect (Qiagen) transfection reagent according to the manufacturer’s instructions. A 10 nM AllStars negative control (miR-ctrl, Qiagen) was used. Efficient miR-30a-3p and miR-30e-3p expression was determined by RT-qPCR (Qiagen) using the StepOne Plus real-time PCR system (Applied Biosystems, Waltham, MA, USA). When indicated, cells were treated with 4% [*v*/*v*] Noggin (U-Protein Express B.V., Utrecht, The Netherlands) or 10 µM A8301 (Sigma-Aldrich). THP-1 monocytes were treated with 50 nM Phorbol 12-myristate 13-acetate (PMA) (PeproTech, Rocky Hill, NJ, USA) to differentiate monocytes into M0 macrophages.

### 4.3. Patient-Derived Tumoroid Culture and Transfection

The study was approved by the Scientific Committee of the tumor bank and the Department of Pathology at the CHU Strasbourg-Hautepierre (Strasbourg, France). Patients signed their informed consent. Tumor extractions were carried out in the Department of Cervicofacial Surgery at the CHU Strasbourg-Hautepierre (France). The resected pieces were histologically diagnosed in the Department of Pathology of the CHU Strasbourg-Hautepierre (France). Tumoroids were extracted from resected tumors following the protocol developed by Driehus et al. [64] and cultured in advanced DMEM/F12 supplemented with GlutaMax, Penicillin/streptomycin, 10mM HEPES, 10 µM Y-27632 (Euromedex, Mundolsheim, France), 0.5 µg/mL Capsofungin (Sigma), 1× B27 supplement (Thermo Fisher Scientific, Waltham, MA, USA), 1.25 mM N-acetyl-L-cysteine (Sigma-Aldrich), 10 mM Nicotinamide (Sigma-Aldrich), 500 nM A8301 (Sigma-Aldrich), 0.3 µM CHIR99021 (Sigma-Aldrich), 50 ng/mL human EGF (PeproTech), 10 ng/mL human FGF10 (PeproTech), 5 ng/mL human FGF2 (PeproTech), 1 µM Prostaglandin E2 (Bio-techne, Minneapolis, MN, USA) and 1 µM Forskolin (Bio-techne), 4% [*v*/*v*] RSPO3-Fc fusion protein conditioned medium (ImmunoPrecise, Utrecht, The Netherlands), and 4% [*v*/*v*] Noggin-Fc fusion protein conditioned medium (ImmunoPrecise). Quality control of tumoroids was performed by histological analysis. Tumoroids were plated at 2500 cells/10 µL of 70% Cultrex UltiMatrix reduced growth factor basement membrane extract (Bio-Techne, Rennes, France) in 24-well plates.

Tumoroids are transfected according to a protocol adapted from Lian et al. [21]. Briefly, cells were plated at a density of 10,000 cells/200 µL/well in 96-well prime surface 3D U plates (SBio, Neuss, Germany) and transfected for 6 h with 100 nM miR-30a-3p, 100 nM miR-30e-3p, or 100 nM miR-ctrl using HiPerFect transfection reagent (Qiagen). After 6 h, cells were collected, centrifuged at 430g for 10 min, and plated at 10,000/40 µL drops composed of 70% Cultrex UltiMatrix reduced growth factor basement membrane extract (Bio-Techne) in 24-well plates. Efficient miR-30a-3p and miR-30e-3p expression was determined by RT-qPCR (Qiagen) using the StepOne Plus real-time PCR system (Applied Biosystems, ThermoFisher Scientific, Illkirch, France). The number of tumoroids and their size was monitored by imaging at 4× and 20× magnification via an Evos XI Core microscope (ThermoFisher Scientific).

### 4.4. Real-Time Quantitative PCR of miRNA on Human Samples

miRNAs were extracted from frozen tumor tissues using miRNeasy kit (Qiagen), according to the manufacturer’s instructions. The integrity of the extracted RNA was verified on an Agilent 2100 Bioanalyser (Agilent Technologies, Palo Alto, CA, USA). RNA concentrations were measured using a ND-1000 NanoDrop spectrophotometer (Labtech, Palaiseau, France). A total of 1 µg of extracted RNA was used for cDNA synthesis using miScript II RT kit (Qiagen), according to the manufacturer’s instructions. A total of 2.5 µL of diluted cDNA corresponding to 50 ng of reverse-transcribed RNA was analyzed with QuantiTect SybrGreen PCR Master Mix (Qiagen), in duplicate, using the LightCycler 480 real-time PCR system (Roche, Meylan, France). qRT-PCR data were analyzed using LightCycler 480 software (Roche, version 1.5). Ct levels were normalized to the geometric mean of the Ct values of 2 internal controls (housekeeping genes): Let7a (5′-TGAGGTAGTAGGTTGTATAGTT-3′, Invitrogen) and RNU44 (5′TGCTGACTGAACATGAAGGTCT-3′, Invitrogen, ThermoFisher Scientific). Primers for miR-30a-3p (HS_miR-30a-3p miScript primer assay, MIMAT0000088; 5′CUUUCAGUCGGAUGUUUGCAGC) and miR-30e-3p (HS_miR-30e-3p miScript primer assay, MIMAT0000693; 5′CUUUCAGUCGGAUGUUUACAGC) were purchased from Qiagen.

### 4.5. Real-Time Quantitative PCR of miRNA and mRNA on Cell Lines and Tumoroids

RNA and miRNAs were extracted from cells or tumoroids using miRNeasy Mini Kit (Qiagen) according to the manufacturer’s instructions. RNA concentrations were measured using an ND-1000 NanoDrop spectrophotometer (Labtech, Palaiseau, France). A total of 1 µg of extracted miRNA/RNA was used for cDNA synthesis using miScript II RT kit (Qiagen, for miRNAs) or iScript Reverse Transcription SuperMix (BioRad, Hercules, CA, USA, for mRNA), respectively. cDNA was analyzed with miScript^®^ SYBR^®^ Green PCR kit (Qiagen) or Fast SYBR™ Green (Applied Biosystem, Thermo Fisher Scientific, Waltham, MA, USA) in duplicate, using the StepOne real-time PCR system (Applied Biosystem). qRT-PCR data were analyzed using StepOne Plus software (Applied Biosystems, version 2.3). The relative expression of each target was calculated using the relative quantification method (2-∆∆CT) with RNU44, Let7, or miR-103-3p for miRNA experiments or RNA18S (Invitrogen) for mRNA experiments as internal controls. The following primer pairs were obtained from Qiagen: GADD45, ACVR1, BMPR2, LTBP2, BNC1, STBN1, CRLF1, TGFB1, TGFBR1, DPYSL3 (see Supplementary Table S2).

### 4.6. Western Blot on Cell Lines and Tumoroids

Cells were lysed (1% Triton-X100, NaF 100 mmol/L, NaPPi 10 mmol/L, Na_3_VO_4_ 1 mmol/L in PBS, supplemented with Complete anti-protease cocktail; Roche) for 20 min at 4 °C and sonicated. A total of 20 µg of protein was separated by SDS-PAGE (4–20% TGX-denaturing gels, BioRad) and transferred to PVDF membranes (Amersham, Sigma-Aldrich, St Quentin Fallavier, France). Blots were probed with TGFBR1 (Abcam, Cambridge, UK ab235178, 1/1000), cleaved PARP (#9541, 1/1000, Cell Signaling, Danvers, MA, USA), cleaved caspase-7 (#9491, 1/1000, Cell Signaling), BMPR2 (ab130206, 1/1000, Abcam), and GAPDH (MAB374, EMD Millipore, Burlington, MA, USA). Proteins were visualized with enhanced chemiluminescence using the LAS4000 microscopy imaging system, and densitometry analysis was performed using ImageJ Software (National Institutes of Health, Bethesda, MD, USA, https://imagej.nih.gov, 1.53t).

### 4.7. Spheres Evasion Assay

After treatment or transfection, 500,000 cells were suspended in 1ml of regular culture medium supplemented with 20% methylcellulose. Spheroids were formed using the hanging-drop culture method. Briefly, drops of 20 µL cell suspension were placed onto the lids of 60 mm dishes, which were inverted over the dishes. Dishes were cultured in humidified chambers (containing PBS) for 48 h to allow the formation of round aggregates. Spheroids were seeded in 24-well plates (4 spheres/well) for 24 h to allow the evasion of cells from attached spheres. When indicated, 4% Noggin [*v*/*v*] or 10 µM A8301 were added to the culture medium. Pictures were taken using the Evos XI Core microscope (AMG, Thermo Fischer Scientific) with 10× magnification. The evasion area (total area − (minus) sphere area) was calculated using ImageJ software, and the results are expressed in arbitrary units of pixels.

### 4.8. Clonogenic Survival Assay

After transfection or treatments, cells were seeded (500 cells/2 mL/well for CAL27 and SCC9 and 1000 cells/2 mL/well for CAL33) in 6-well plates and allowed to grow for 10 days. Cells were stained with crystal violet at 0.1% (Sigma-Aldrich, St Quentin Fallavier, France). Colonies were counted to determine the plating efficiency (PE) and the surviving fraction (SF). PE = number of surviving cells/number of cells plated. SF = PE of the experimental group/PE of the control group.

### 4.9. Immunohistochemistry on Tumoroids

Expression of Keratin 34BE12, p40, and p63 was evaluated by immunohistochemical (IHC) analysis using a Ventana Autostainer Automat (Ventana Medical Systems, Roche Tissue Diagnostics) at the Department of Pathology (Strasbourg University Hospital). Slides were prepared from formalin-fixed paraffin-embedded tumor specimens and corresponding tumoroids. Slides were stained for Keratin 34BE12 (M0630, clone 34BE12, 1/50, DAKO Agilent), p40 (RP 136-05, 1/200, Diagnostic Biosystems Pleasanton, Pleasanton, CA, USA), and p63 (790-4509, Ventana, Roche) according to the manufacturers’ instructions. Signals were revealed with the ultraView Universal DAB Detection Kit (Ventana Medical Systems, Roche Tissue Diagnostics, Oro Valley, AZ, USA), according to the manufacturer’s instructions. All images were acquired with an Olympus BX60 with 20× or 40× objectives. Contrasts were uniformly adjusted on all images with Photoshop (Adobe) software (CS2, version 9).

### 4.10. Immunofluorescence of Cells

After transfection or treatments, cells were seeded in a Nunc Lab-Tek II CC2 8-well Chamber Slide System at a density of 10,000/well and cultured for 2 days. Cells were fixed with 4% paraformaldehyde or with ice-cold methanol for 15 min. Samples were blocked in PBS/5% BSA/0.3% Triton X-100 for 1 h and incubated overnight at 4 °C with TGFBR1 (ab235178, 1/100, Abcam) or BMPR2 (ab130206, 1/100, Abcam). After washing in PBS, cells were incubated with Alexa Fluor™ 568 goat anti-mouse or anti-rabbit secondary antibodies (1/500, Life Technologies, Thermo Fisher Scientific). Slides were mounted using Fluoromount-G medium (#00-4958-02; Thermo Fischer Scientific). Images were acquired using a LEICA DMI 4000B confocal microscope (Leica Microsystems SA, Nanterre, France) with a 63× magnification oil-immersion objective. The intensity of fluorescence was measured using ImageJ software.

### 4.11. Immunofluorescence on Tumoroids

Following recovery, tumoroids were fixed in PFA 4% for 20 min and washed in PBS. After a 15 min permeabilization step in PBS/0.1% Tween-20 and a 60 min blocking step in PBS/0.1% Triton X-100/2% BSA/5% NGS, tumoroids were incubated overnight at 4 °C with BMPR2 primary antibody. After washing in PBS/0.1% Triton X-100/0.2% BSA, cells were incubated for 3 h at room temperature with appropriate secondary antibodies (Life Technologies; dilution 1/500) and DAPI (#D9542; Sigma-Aldrich, St Quentin Fallavier Cedex, France; 1 µg/mL). After washing twice in PBS/0.1% Triton X-100/0.2% BSA and twice in PBS, the slides were mounted using FUnGI medium (50% [*v*/*v*] glycerol, 9.4% [*v*/*v*] dH_2_O, 10.6 mM Tris base, 1.1 mM EDTA, 2.5 M fructose and 2.5 M urea). Images were acquired using a LEICA TCS SPE II confocal microscope (Leica Microsystems SA), with a 20× magnification objective, and analyzed with ImageJ software or Imaris software (Imaris ×64 9.3.1—22 May 2019).

### 4.12. Phagocytosis Assay

CAL27, CAL33, or SCC9 was transfected with miR-30a-3p or miR-30e-3p. A total of 72 h after transfection, particles from the supernatant were removed by centrifugation, and this medium was either frozen or used immediately. We called this medium the conditioned medium. THP-1 cells were seeded at a density of 100,000 cells/well on a Nunc Lab-Tek II CC2 8-well Chamber Slide System and differentiated into M0 macrophages with 50 nM of PMA. After 24 h, CAL27, CAL33, or SCC9 conditioned medium was added to THP-1 cells (70% conditioned medium and 30% RPMI medium). A total of 24 h later, 1 µM of CytoTrace™ Green CMFDA (CliniSciences, Nanterre, France) was added to THP-1 and 1 µM of CellTracker™ Deep Red Dye (Invitrogen, Thermo Fisher) was added on transfected CAL27, CAL33, or SCC9 for 30 min at 37 °C. Tumor cells were then added on THP-1 cells at a 1:1 ratio for 4 h. When mentioned, 10 µg/mL of CD47 antibody (B6H12, Thermo Fisher Scientifics) was added to the co-culture as well. Cells were fixed with PFA 4% for 10 min and washed 3 times with PBS. Slides were mounted using Fluoromount-G medium (#00-4958-02; Thermo Fischer Scientific) and then observed using a LEICA DMI 4000B confocal microscope (Leica Microsystems SA) with a 20× or 63× magnification oil-immersion objective. The percentage of phagocytosis was calculated as the number of THP-1 phagocytosing HNSCC cells/total number of THP-1 × 100.

### 4.13. Bioinformatics Analyses of Target Genes

Expression data of miR-30a-3p (MIMAT0000088) and miR-30e-3p (MIMAT0000693) were selected from a cohort of 211 patients from the TCGA-HNSC project of the databank cBio Cancer Genomics Portal of TCGA (https://portal.gdc.cancer.gov/). Patients were sorted according to the localization of their tumors (oropharynx, hypopharynx, and oral cavity) and the availability of follow-up. Patients who died before 50 days of the follow-up and patients who were HPV+ were discarded from the analysis. All the statistics were performed using R software (R version 4.0.3). The distribution of miRNAs expression was divided into two populations of low and high expression based on the median or the third quartile. Correlation was determined between the expression of 442 genes and miRNAs, and the threshold was fixed to the absolute value of 0.2. Matching sequence between miR-30a-3p or miR-30e-3p and their target genes were performed using TargetScan release version 8.0 (https://www.targetscan.org/). The interaction between target proteins was analyzed using STRING program (Search Tool for the Retrieval of Interacting Genes/Proteins) version 11.5 (https://string-db.org/).

### 4.14. Statistical Analysis

Data are expressed as mean ± SEM and analyzed using GraphPad Prism version 5 (GraphPad Software, San Diego, CA, USA). Differences between groups were analyzed using a non-paired *t*-test, and *p* < 0.05 was considered statistically significant.

Survival analyses: Overall survival (OS), locoregional relapse-free survival, and metastasis-free survival were estimated using the method of Kaplan–Meier. Inferential analysis of qualitative variables was performed using a log-rank test, and comparison of quantitative variables was performed using the Cox model. Multivariate analyses were performed using all variables that were statistically significant in univariate analyses or according to clinical importance. Stepwise regression was performed with backward selection to identify variables of potential prognostic relevance. *p* < 0.05 was considered significant. All analyses were performed using R 3.1.0 software and the survival package. Cut-off values were set as the average ± SEM.

miR-30a-3p and miR-30e-3p cut-off thresholds: The thresholds were based on ROC analysis for defining high and low miR-30e-3p and were determined empirically to differentiate most significantly between miR-30a-3p high and low groups based on survival data. The threshold was fixed as the first quartile for miR-30a-3p.

## 5. Conclusions

In conclusion, our study showed that miR-30a-3p and miR-30e-3p act as tumor suppressors in HNSCC cells and patient-derived tumoroids. They identify subgroups of LA-HNSCC patients with different prognoses, making them good candidates to become tissue and/or circulating biomarkers predictive of survival and relapse. By targeting members of the TGF-β family, they may emerge as an alternative to anti-TGF-β signaling drugs to use alone or in combination with immune or macrophage checkpoint inhibitors.

## Data Availability

Not applicable.

## Supplementary Materials

The following supporting information can be downloaded at: https://www.mdpi.com/article/10.3390/ijms241311178/s1.
