# Supplementary material for: Tumor-Suppressive and Immunomodulating Activity of miR-30a-3p and miR-30e-3p in HNSCC Cells and Tumoroids
Source: Int J Mol Sci. 2023 Jul 6;24(13):11178. doi: 10.3390/ijms241311178 (PMC10342301; doi:10.3390/ijms241311178)
Supplement: Supplementary file 1 [file ijms-24-11178-s001.zip › ijms-2458952-supplementary.pdf]

Supplementary Data S1: After transfection or treatments, cells were seeded (2,000 cells/200µl/well for CAL27 and SCC9 and 8,000 cells/200µl/well for CAL33) in 96-well plates at 37°C. Confluence (shown in orange) of CAL27, CAL33 and SCC9 cell lines overexpressing miR-30a-3p or miR-30e-3p was monitored up to 7 days. Images are representative of 6-10 experiments.

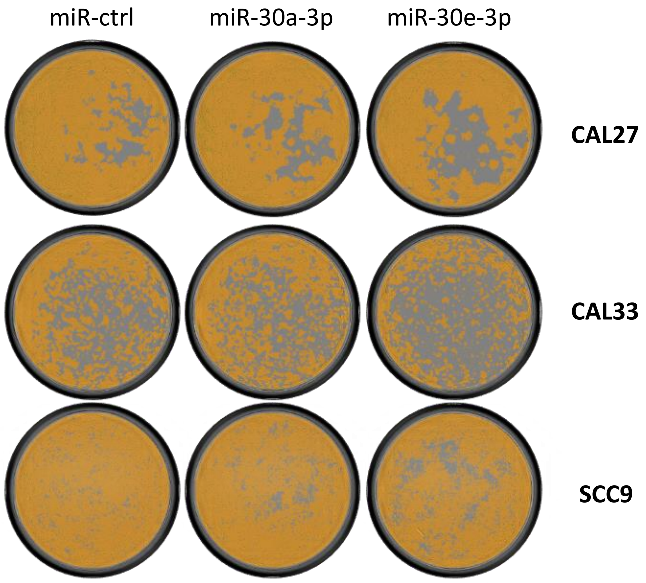

Supplementary Data S2: a. BMPR2 expression was determined by RT-qPCR in M0 polarized THP-1 exposed to CAL27, CAL33, SCC9 overexpressing miR-30a-3p and miR-30e-3p conditioned media (n = 3-4, * p < 0.05). **b**. miR-30a-3p and miR-30e-3p expression was determined by RT-qPCR in M0 polarized THP-1 exposed to CAL27, CAL33, SCC9 overexpressing miR-30a-3p and miR-30e-3p conditioned media (n = 1).

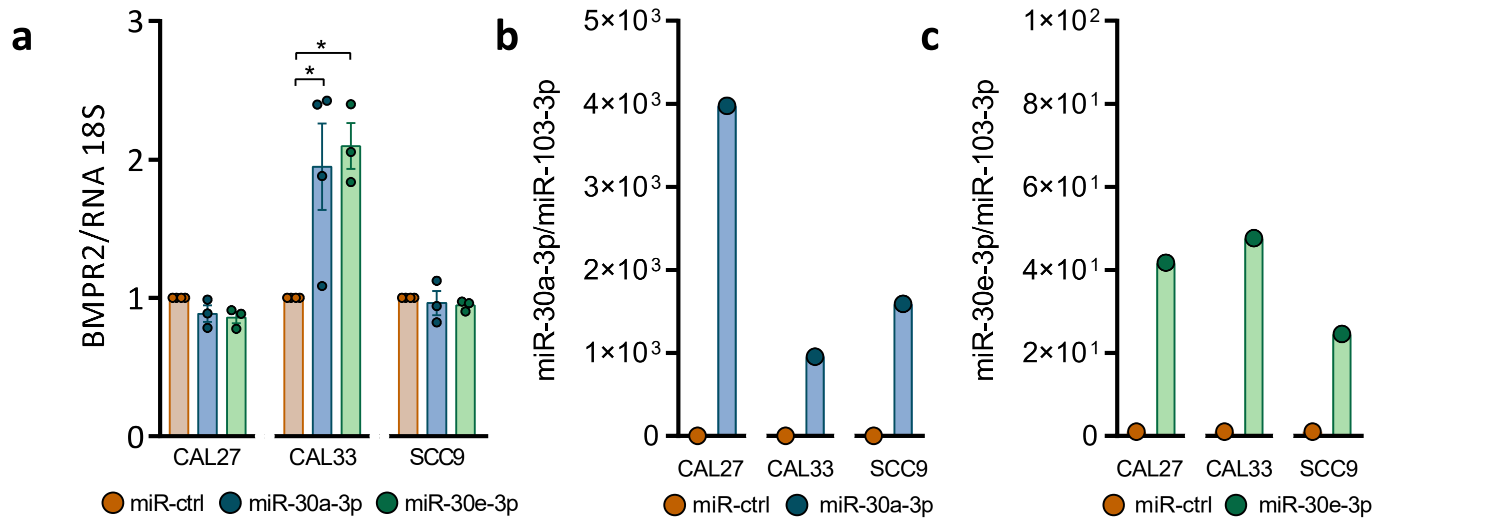

Supplementary Table S1: Clinical characteristics of the Patients

Clinical characteristics of patients		
**Variable**		**All patients**
**Sex — no. (%)**		
Male		90 (82)
Female		20 (18)
**Age**		
Median age —yr		57
<65yr — no. (%)		78 (71)
≥65yr — no. (%)		32 (29)
**ECOG — no. (%)**		
0-1		94 (85)
2		10 (9)
≥3		4 (4)
missing		2 (2)
**Karnofsky score**		
Median score		90
Interquartile range		90-100
<80yr — no. (%)		14 (13)
≥80yr — no. (%)		94 (85)
missing		2 (2)
**Charlson Comorbidity Index — no. (%)**		
Median score		4
0		0
1-2		14 (13)
3-4		51 (46)
≥5		41 (37)
missing		4 (4)
**Smoking — no. (%)**		
smoking cessation		46 (42)
active smoking		53 (48)
pack-years means		42
no history of smoking		5 (5)
missing		6 (5)
**Alcohol consumption — no. (%)**		
history of alcohol addiction		72 (65)
missing		15 (14)
**Primary tumor site — no. (%)**		
Oral cavity		27 (25)
Oropharynx		26 (24)
Hypopharynx		29 (26)
Overlap between several tumor sites		28 (25)
**TNM classification — no. (%)**		
**T**	T1	4 (4)
	T2	38 (35)
	T3	45 (41)
	T4	23 (20)
**N**	N0	15 (14)
	N1	8 (7)
	N2a	7 (6)
	N2b	41 (37)
	N2c	24 (22)
	N3	15 (14)
**Histologic type and characteristics — no. (%)**		
Well differentiated		16 (14)
Modertely differentiated		63 (57)
Poorly differentiated		26 (24)
Indifferentiated		1 (1)
Squamous-cell carcinoma not specified		4 (4)
Lymphovascular emboli		24
Perineural invasion		11
Capsular rupture of the lymph nodes		80
Positive surgical margins		51
**First-line treatment — no. (%)**		
Adjuvant radiotherapy alone		37 (33)
Concomitant chemotherapy		69 (63)
Without adjuvant therapy		4 (4)
**Extent of disease — no. (%)**		
Only locoregionally recurrent		18 (16)
Metastatic with or without locoregional recurrence		33 (30)
**Total — no. (%)**		**110**

Supplementary Table S2: Accession numbers and reference positions or sequences (depending of the provider) of primers used in RT-qPCR analysis.

Primer sequence	Accession number	Reference position or sequence
Hsa-miR-30a-3p	MIMAT0000088	5'CUUUCAGUCGGAUGUUUGCAGC
Hsa-miR-30e-3p	MIMAT0000693	5'CUUUCAGUCGGAUGUUUACAGC
Let7a	MI0000060	5’TGAGGTAGTAGGTTGTATAGTT
RNU44	NR_002750	5’TGCTGACTGAACATGAAGGTCT
BMPR2	NM_001204.6	3909
ACVR1	NM_001105.4	1860
TGFBR1	NM_004612.3	1142
LTBP2	NM_000428.2	5359
SPTBN1	NM_003128.2	6443
TGFB1	NM_000660.5	1464
CRLF1	NM_004750.5	1312
RNA18S	NR_003278.3	Forward: 5’-TGTGGTGTTGAGGAAAGCAG<br>Reverse: 5’-TCCAGACCATTGGCTAGGAC
